# Incidence and Sociodemographic Correlates of Psychological Health Problems among Residents of the West Pomeranian Voivodeship during the COVID-19 Outbreak

**DOI:** 10.3390/medicina58020196

**Published:** 2022-01-27

**Authors:** Anna Maria Cybulska, Kamila Rachubińska, Małgorzata Starczewska, Labib Zair, Mariusz Panczyk

**Affiliations:** 1 Department of Nursing, Faculty of Health Sciences, Pomeranian Medical University in Szczecin, Żołnierska 48, 71-210 Szczecin, Poland; anna.cybulska@pum.edu.pl (A.M.C.); malgorzata.starczewska@pum.edu.pl (M.S.); 2 Department of General Surgery and Transplantation, Pomeranian Medical University in Szczecin, Powstańców Wielkopolskich 72, 71-210 Szczecin, Poland; labib@poczta.onet.pl; 3 Department of Education and Research in Health Sciences, Faculty of Health Science, Medical University of Warsaw, Litewska 14/16, 00-581 Warsaw, Poland; mariusz.panczyk@wum.edu.pl

**Keywords:** health, COVID-19, SARS-CoV-2, depression, anxiety, stress, insomnia

## Abstract

*Background and Objectives*: Psychological health problems have become an important topic of consideration for many scientists, because the epidemiology of these disorders is strongly influenced by stressful events such as the SARS-CoV-2 coronavirus pandemic. The aim of this study was to evaluate selected parameters of psychosocial functioning as well as socio-demographic correlates of depression, anxiety, sleep disorders and perceived stress among the residents of the West Pomeranian Voivodeship. *Materials and Methods*: An online questionnaire was completed by 323 participants, in whom the parameters of psychosocial functioning were assessed (symptoms of depression, anxiety, severity of sleep disorders and perceived stress). *Results*: The majority of the respondents (75.2%) scored high on the Perceived Stress Scale, and almost half of the respondents (47.1%) had sleep disorders. A total of 26% of the participants had no depressive symptoms. Age was significantly correlated with the severity of depressive symptoms and sleep disorders. There was a strong correlation between the severity of depression and anxiety (r = 0.76; *p* < 0.0001), a moderate correlation between depression and perceived stress (r = 0.47; *p* < 0.0001) and a strong correlation between depression and sleep disorders (r = 0.651; *p* < 0.0001). *Conclusions*: Age contributed to the severity of depressive symptoms and the occurrence of sleep disorders among the residents of the West Pomeranian Voivodeship during the SARS-CoV-2 pandemic. Some residents of the West Pomeranian Voivodeship showed moderate to severe depressive and anxiety symptoms, as well as high levels of stress and insomnia.

## 1. Introduction

On 31 December 2019, a new coronavirus (COVID-19) disease emerged in Wuhan, Hubei Province, China, which was declared an international public health emergency by the World Health Organization (WHO) [1]. The scale of the pandemic gave rise to concern all over the world not only for the victims, but also for the psychological and socioeconomic consequences. Major changes that took place in the daily functioning of society contributed to increased psychological distress, including symptoms of depression, anxiety, stress, and sleep disorders [2]. The introduced restrictions, isolation, limits on civil liberties, and contact with an ‘unknown enemy’ against which there is no effective defense may have contributed to significant changes in the human psyche, including the development of generalized anxiety, depression, and anxiety symptoms.

There are individuals in society who are particularly vulnerable to stress and who cannot cope with a new and difficult situation. As a result of the restrictions imposed by the COVID-19 pandemic, many people may experience increased anxiety, loneliness or depression. In addition, all are forced to face new and serious challenges, such as financial difficulties. Moreover, the lack of opportunities or difficulties in developing effective methods of coping with stress, resulting, for example, from the lack of social support, may lead to emotional overload, including increased anxiety, sadness, and depressive symptoms. Most often, the conditions developed during a pandemic are an expression of temporary adaptation problems. However, in some people they may be a symptom of serious mental problems, requiring professional psychological care. Particularly prone to this stress are people who feel a real threat to their health or who have lost their jobs [3,4].

COVID-19 poses a threat to the physical health of both infected people and the general public. A Canadian survey demonstrated that 36% of the population was very concerned about the impact of COVID-19 on their health [5]. An online survey in China, based on the EuroQol-5D test assessing health-related quality of life, showed that 19% of the participants experienced physical pain or discomfort [6]. The COVID-19 pandemic, which has also severely affected the mental health of the public, may threaten the bodily integrity and autonomy of an individual, and subsequently result in comorbid psychiatric illnesses manifested by atypical symptoms, such as functional movement disorders [7,8]. 

Mental health is considered the most important condition for a good quality of life. Unfortunately, stressful events are strong adverse environmental factors that predispose people to mental disorders, especially depression [9]. During the COVID-19 pandemic, many people experienced negative emotional reactions; therefore, the National Health Commission issued guidelines to promote psychological crisis intervention targeted at patients, medical professionals, and civilians during the COVID-19 pandemic [10]. 

The aim of this study was to assess the severity of depression, anxiety, sleep disorders, and perceived stress depending on sociodemographic data, as well as to assess correlations between variables related to psychosocial functioning. 

## 2. Materials and Methods

### 2.1. Settings and Design

The study adopted a cross-sectional survey design to assess the immediate psychological response of the residents of the West Pomeranian Voivodeship during the COVID-19 pandemic. An anonymous online questionnaire was used.

Due to the recommendations of the Polish government to minimize contact with other people, potential respondents were invited to take part in the survey electronically. The volunteers completed questionnaires in Polish via an online platform (https://docs.google.com/ accessed on 20 November 2021). The study received a positive opinion from the Bioethics Committee of the Pomeranian Medical University of Szczecin. The study was conducted in accordance with the principles contained in the Declaration of Helsinki. Participation in the study was voluntary and anonymous. The respondents were informed about the purpose of the study and the possibility of resignation and withdrawal of consent at each stage of the study.

The inclusion criteria for the study were: residency in the West Pomeranian Voivodeship; age over 18 years; and informed consent to participate in the study. The project was approved by the Bioethics Committee of the Pomeranian Medical University in Szczecin (KB-0012/25/04/2020/Z).

**Figure 1:**
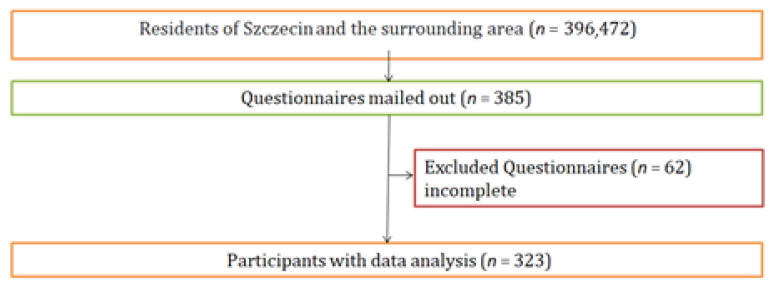
Flowchart of survey participation.

The size of the research sample was established on the basis of statistical data on the population of Szczecin and its vicinity aged 18–64 in 2020 [11]. The confidence level was set at 95%, the maximum error at 5%, and the estimated fraction size at 0.5. The total number of respondents who qualified for the study was 384. Finally, 323 respondents who correctly completed the questionnaires were included in the further analysis (Figure 1). 

### 2.2. Research Instruments

The set of questionnaires was created after previous analysis of the literature on the impact of COVID-19 on the mental health of the population. The following standardized questionnaires were used: 

Generalized Anxiety Disorder-7 (GAD-7): a screening tool used to determine feelings related to generalized anxiety disorder (GAD) [12]. 

Patient Health Questionnaire-9 (PHQ-9): a screening tool for depression, developed on the basis of the diagnostic criteria for depression included in the Diagnostic and Statistical Manual of Mental Disorders (DSM-IV) [13]. 

Athens Insomnia Scale (AIS): an eight-item scale that allows quantitative measurement of insomnia symptoms based on the ICD-10 criteria. The scale consists of eight statements regarding insomnia symptoms [14].

Perceived Stress Scale (PSS-10): an instrument used to assess the severity of stress related to the situation of the respondent in the last four weeks, in the context of subjective feelings and problems in personal life [15].

Demographic data (age, education, place of residence in the last 14 days, marital status, employment status, parental status, and household size), physical symptoms in the last 14 days (fever, chills, headache, muscle aches, cough, difficulty breathing, dizziness, sore throat, and persistent fever), history of exposure to COVID-19 (close contact with a person with confirmed COVID-19), and additional information required in relation to COVID-19 was collected using the authors’ questionnaire.

### 2.3. Statistical Analysis

Descriptive statistics were calculated for sociodemographic characteristics, physical symptoms, contact history variables, and anxiety-related variables. The response rate was calculated based on the number of respondents per the total number of responses to the question. Analysis of quantitative variables (expressed as numbers) was performed by calculating the mean, standard deviation, median, quartiles, as well as minimum and maximum values. Student’s *t* tests and ANOVA were used in this study. 

All calculations were performed using STATISTICA TM 13.3 (TIBCO Software, Palo Alto, CA, USA). The level of statistical significance was set at *p* < 0.05.

## 3. Results

### 3.1. Characteristics of the Respondents 

The study sample consisted of 323 individuals who correctly completed the questionnaires. The mean age was 35.4 years (SD = 10.9). The vast majority of the respondents were women: 79.3%; people in a formal relationship constituted 47.4%, those with higher education: 78.1%; and those living in a city with more than 100,000 inhabitants: 57% (Table 1). 

A total of 64.7% of the respondents had not been diagnosed with any chronic disease; 57.3% assessed their health as good; and 26.3% as very good. Only 9.6% of the subjects were in quarantine due to COVID-19, and 8.4% had a medical consultation in the past 14 days. Part of the participants (38.7%) had no contact with other people except for family members within four weeks of completing the survey questionnaire, whereas 31% had close contact and 20.4% had indirect contact with a person with confirmed COVID-19 infection. 

Analysis was performed on depressive symptoms (according to the PHQ-9), anxiety (according to the GAD-7), sleep disorders (according to the AIS), and perceived stress (according to the PSS-10) among residents of the West Pomeranian Voivodeship during the SARS-CoV-2 pandemic. 

For the Perceived Stress Scale, the mean score was 7.3 out of 10, and most of the respondents (75.2%) scored high. Mild anxiety was observed in 34.06%, moderate anxiety in 18.89%, and severe anxiety in 17.65% of the respondents. Almost half of the respondents (47.1%) suffered from sleep disorders. A total of 26% of the subjects had no depressive symptoms, while 28.79% showed mild, 23.53% moderate, 14.55% moderately severe, and 7.12% severe symptoms of depression (Table 2 and Table 3). 

### 3.2. Analysis of the Relationship between Sociodemographic Variables (Age, Education, Place of Residence, Marital Status, Parental Status) and the Severity of Anxiety, Depression, Perceived Stress, and Insomnia among the Residents of the West Pomeranian Voivodeship during the SARS-CoV-2 Pandemic 

Age was found to significantly correlate with the severity of depression and the presence of sleep disorders. There were no statistically significant correlations between age and the other scales (Table 4). 

Analysis of the data revealed statistically significant relationships between sex and the selected parameters of psychosocial functioning: depression, anxiety, and stress (Table 5). 

Parental status was statistically significantly related to the severity of depression (PHQ-9). No other statistically significant correlations were observed between parental status and the other scales (Table 6). 

Analysis of the influence of other sociodemographic variables (education, place of residence, marital status) on levels of anxiety, depression, perceived stress, and insomnia among residents of the West Pomeranian Voivodeship during the SARS-CoV-2 pandemic did not reveal any statistically significant correlations (Supplementary material Tables S1–S3). 

Analysis of the data demonstrated a strong, positive correlation between the severity of depression and the severity of anxiety (r = 0.76, *p* < 0.001) and the occurrence of sleep disorders (r = 0.65, *p* < 0.001), as well as a moderate positive correlation between depression and stress (r = 0.45, *p* < 0.001). Moreover, the level of anxiety strongly correlated with sleep disorders (r = 0.53, *p* < 0.0001) and the level of stress (r = 0.50 *p* < 0.001). The severity of stress showed a moderate correlation (r = 0.32, *p* < 0.001) with sleep disorders (Table 7).

## 4. Discussion

The studies conducted so far among pandemic-affected populations clearly indicate its significant impact on their mental health [16]. There are many factors that predispose to mental illness [5,6]. The pandemic entails the need for quarantine and isolation, which are also among the risk factors with a psychological impact [4,7], as is worrying about the health of family, friends and acquaintances [17].

According to the pre-pandemic reports, symptoms of generalized anxiety disorder were found in about 9.6% of the Polish population, more often in women than in men [18]. On the other hand, Lubecka et al. [19] showed that the point criteria for anxiety disorders were met by 11.2% of the respondents, while a depressive episode was diagnosed in 14.4% [19].

The mental health of the general public is at greater risk compared to the situation before the outbreak [16,20]. Studies conducted during the previous SARS-CoV-1 epidemic showed that people who were directly affected (e.g., by quarantine) had psychiatric symptoms that lasted for several months after the epidemic ended [21], which may indicate that long-term SARS-CoV-2 consequences should also be expected. Our own study demonstrates anxiety of varying severity in the majority of the residents of the West Pomeranian Voivodeship. Similar results were obtained by Babicki et al. [8], who reported anxiety symptoms of different severity in about 75%, severe anxiety symptoms in 23%, and the features of generalized anxiety disorder in 44% of the respondents. Such a high percentage of positive results may be due to restrictions and the feeling of helplessness and powerlessness in the fight against the coronavirus. The possibility of direct interpersonal contact, which, according to specialists, is necessary to maintain a person’s mental balance, was significantly limited [22,23]. Worldwide reports on the intensity of anxiety in populations severely affected by the COVID-19 pandemic, as in Poland, showed a significant increase in anxiety compared to the state before 2019. An analysis of a survey conducted with the use of the GAD-7 questionnaire among Chinese students showed that 25% of the respondents obtained a result indicating anxiety symptoms of varying severity [24]. A study of 1210 Chinese residents indicated the presence of anxiety symptoms in 36% of the subjects [25]. In addition, 51% of Iranians showed anxiety during the COVID-19 pandemic [26].

In our research, half of the respondents had moderate to severe depressive and anxiety symptoms, and 65% had moderate to severe anxiety symptoms. Our findings support the results obtained by Wang et al. [27] in their study based on the Impact of Event Scale-Revised (IES-R) and the Depression, Anxiety and Stress Scale (DASS-21), which confirmed a significant contribution of the COVID-19 epidemic to mental deterioration. Most of the respondents indicated a moderate or significant psychological impact of the epidemic and had major depressive symptoms. Among women, the impact of the epidemic on psychological functioning was more pronounced, as demonstrated by both the IES-R and the DASS-21 scales [27]. Another large population-based study conducted in China provided evidence that more than one-third (~35%) of the subjects experienced psychological distress ranging from mild to moderate (>29%) and severe (>5%) [28]. Verma et al. observed that the majority of the respondents in their study exhibited depressive and anxiety symptoms of varying severity [29]. Female sex, young age, higher education, student status, and having certain physical symptoms (muscle pain, dizziness, back pain) were significantly related to higher levels of stress, anxiety and depression [28,30,31]. Wang et al. also showed the negative impact of social media and massive amounts of information, as well as poor availability of personal protective equipment and accessibility to health care, on the level of psychological distress. Anxiety and depression are two different mental health disorders, and their biological development mechanisms are not identical [32].

As the data show, sex significantly differentiated the study group in terms of both anxiety and depressive symptoms. Women declared significantly more concerns about everyday life in the pandemic than men, and also showed significantly more depressive symptoms. However, due to large disparities between the studied groups of women and men, despite the use of appropriate tests, the observed differences should be approached with caution.

These results are consistent with those obtained by other authors, who also concluded that women are more likely to experience depression than men [33]. Furthermore, research conducted in Turkey during the pandemic indicates that women are more likely to suffer from depression than men [34]. A study conducted in China shows that women experience anxiety symptoms more often than men during a pandemic [30]. As shown by a study conducted in Poland at the beginning of May 2020, with a comparable number of women (49.7%) and men (50.3%), the incidence of both severe depressive symptoms and generalized anxiety is similar in women and in men [35]. Considering the above, and with some caution, due to the large disparities in the size of the studied groups of women and men, it can be concluded that women experience higher levels of anxiety and depressive symptoms during a pandemic.

In our study, almost half of the respondents developed sleep disorders. Roy et al. [36], based on their cross-sectional study conducted in India at the beginning of 2020, indicated that over 80% of the participants experienced preoccupation with COVID-19, and almost half felt panic about media reports. 

Physical symptoms and poor self-rated health were significantly associated with a higher incidence of post-traumatic stress disorder and symptoms of stress, anxiety, and depression [37,38]. Both sleep problems [31] and suicidal thoughts [39] are serious mental health problems in the COVID-19 era. It can be hypothesized that deterioration in mental and physical health is significantly related to sleep problems and suicidal thoughts, which have become more common during the COVID-19 pandemic. 

Ozamiz-Etxebarria et al. [40] found that women had more depressive, anxiety and stress-related symptoms than men, as measured by the DASS-21. The lowest severity of symptoms was recorded in the oldest age group (61 years and older). The authors suggest a link between this distribution of results and the additional stress experienced by young adults—mostly students—due to remote learning.

We found that age and parental status had an impact on the severity of depression, and age influenced the occurrence of sleep disorders. Research into the relationship between demographic characteristics and physical and mental health during the COVID-19 pandemic has revealed mixed results. One study provided evidence that aging people had a greater risk of physical pain or discomfort and depression or anxiety [14], while another research report revealed that young people were more likely to complain of mental health problems during the COVID-19 pandemic [18,23,31]. Moreover, several studies have confirmed that women are more likely to report poor mental health during the COVID-19 pandemic than men [18,23,25,32]. However, sex as a determinant of physical health during the COVID-19 pandemic has not been investigated. Further research is needed to determine if demographic factors are related to deterioration in physical and mental health during the COVID-19 pandemic. 

The results of our research indicate a deterioration in the mental health of the residents of the West Pomeranian Voivodeship, but this problem affects people all over the world. A study conducted in China during the pandemic showed a significant decline in the well-being and mental health of the general population. A large population questionnaire-based study, performed using the Impact of Event Scale-Revised (IES-R) and the Depression, Anxiety and Stress Scale (DASS-21) demonstrated a significant contribution of the COVID-19 epidemic to psychological decline: 53.8% of the subjects reported a moderate or significant impact, 21.7% a mild impact, and only 24.5% experienced only a minimal psychological impact of the epidemic [27]. The results of the depression subscale of the DASS showed that 12.2% of the respondents experienced symptoms of moderate depression, and 4.3% had symptoms of severe depression. On the anxiety subscale, 7.5% of the subjects obtained results reflecting mild anxiety, 20.4%―moderate anxiety, and 8.4%―severe or very severe anxiety. According to the results of the stress subscale, 24.1% of the participants experienced mild stress, 5.5%―moderate stress, and 2.6%―severe stress. 

An Italian study showed that sociodemographic variables explain about 30% of the variance in the results for anxiety, depression and stress [37]. This cross-sectional study used the DASS-21 and the Personality Inventory for DSM-5―Brief Form (PID-5-BF). For depressive symptoms, 17% of the subjects scored high and 15.4% scored very high; sociodemographic variables explained only about 9% of the variability in the results. Severe anxiety symptoms were declared by 7.2% of the respondents, and very severe by 12.6%. Young age, female sex, a family member with COVID-19, history of stressful events, and medical problems were associated with higher levels of anxiety. A total of 14.6% of the respondents declared a high level and 12.6%―a very high level of stress. Young age, female sex, the necessity of going to work (no possibility to work remotely), a history of stressful events and medical problems, and having a friend with a confirmed COVID-19 infection were associated with higher levels of stress. It can be assumed that most cases of elevated distress were related to adaptation difficulties and the trauma of the pandemic [36].

The analysis of the literature also confirms the results obtained. The groups of respondents in which high levels of anxiety, stress and depressive symptoms were observed, that is mainly young people, women, and people with offspring are described as those at higher risk of experiencing various types of mental health disorders as a consequence of the pandemic and the restrictions introduced in response to it [41,42,43,44].

## 5. Conclusions

- Our study demonstrated that during the pandemic, some residents of the West Pomeranian Voivodeship experienced moderate to severe depressive and anxiety symptoms, as well as sleep disorders and high levels of stress.
- During the COVID-19 pandemic, a negative impact on mental health was demonstrated in the study group of residents of the West Pomeranian Voivodeship, mainly including the risk of depression, anxiety or post-traumatic stress disorder. This was especially true for the elderly and people with parental status, as these respondents had experienced an increase in depression and a greater risk of sleep disorders.
- It has been shown that stress exposure is a risk factor for depressive symptoms and anxiety among the respondents; therefore, it is important to carefully monitor mental health during a pandemic so that preventative measures can be taken as early as possible.

## 6. Practical Recommendations

We divide recommendations by general lifestyle during the COVID-19 pandemic:

- Regular and sufficient sleep, regular and healthy meals, drinking sufficient fluids, and taking care of personal hygiene are essential not only for maintaining good physical health, but also for improving mental well-being.
- Taking up physical activity is not only essential for maintaining a healthy body, but it also helps to improve mood by lowering levels of stress hormones and stimulating the production of endorphins, and it has a beneficial effect on immune function.
- Using relaxation and stress reduction techniques (e.g., reading, writing, listening to music, meditation, autogenic training, and mindfulness exercises) can help you stay healthy and be aware of your emotions. When dealing with difficulties, talking openly about emotions with loved ones, asking for help, and feeling social support can be effective in reducing stress and anxiety.
- Enjoying interpersonal relationships: remember to meet regularly with your loved ones. Family time can include important conversations, party games or sports, eating meals and doing household chores together.
- Follow the WHO recommendations to stay up to date on pandemic and public health information by using credible news sources (e.g., watching reputable news programs once or twice a day) and limiting exposure to non-informed media. This can promote balanced and informed thinking about a pandemic.

## Figures and Tables

**Table 1:** Sociodemographic variables.

Variables (*N* = 323)		*n*	%
Gender	Female	256	79.3
	Male	*67*	20.7
Marital status	Formal relationship	153	47.4
	Informal relationship	78	24.1
	Single/divorced/widowed	92	28.5
Education	Secondary	57	17.6
	post-secondary	14	4.3
	Higher (I)	99	30.7
	Higher (II)	141	43.7
	Higher (III)	12	3.7
Place of residence	village	67	20.7
	city with up to 10,000 people	16	5.0
	city with 10,000–100,000 people	56	17.3
	city with over 100,000 people	184	57.0
Children	No	160	49.5
	Yes	155	48.0
	Pregnancy	8	2.5

*N*, whole cohort size; *n*, number of; %, percent.

**Table 2:** The psychosocial functioning (anxiety, depression, insomnia, stress) of the respondents.

Severity Category (Points)						
Variables	M	SD	Me	IQR/2	Min-Max	CV [%]
AIS	7.56	4.51	7.0	3.5	0.0–24.0	59.6
GAD-7	15.34	5.95	14.0	4.5	7–28	38.8
PHQ-9	18.30	6.35	18.0	4.5	9–36	34.7
PSS-10	7.30	1.60	8.0	0.5	0.0–10.0	21.9

M—mean; SD—standard deviation; Me—median; IQR/2—interquartile range; Min—minimum; Max—maximum; CV—coefficient of variation; GAD-7—Generalized Anxiety Disorder-7; PHQ-9—Patient Health Questionnaire-9; AIS—Athens Insomnia Scale; PSS-10—Perceived Stress Scale.

**Table 3:** The psychosocial functioning (anxiety, depression, insomnia, stress) of the respondents.

Severity Category			
Variables (Points)		*n*	%
AIS	no insomnia disorder	171	52.9
	yes >8 points	152	47.1
GAD-7	no anxiety disorder (0–4 points)	95	29.41
	mild anxiety disorder (5–9 points)	110	34.06
	moderate anxiety disorder (10–14 points)	61	18.89
	severe anxiety disorder (15–21 points)	57	17.65
PHQ-9	no depression (0–4 points)	84	26.01
	mild depression (5–9 points)	93	28.79
	moderate depression (10–14 points)	76	23.53
	moderately severe depression (15–19 points)	47	14.55
	severe depression (20–27 points)	23	7.12
PSS-10	low stress (1–4 sten)	18	5.6
	medium stress (5–6 sten)	62	19.2
	high stress (7–10 sten)	243	75.2

GAD-7—Generalized Anxiety Disorder-7; PHQ-9—Patient Health Questionnaire-9; AIS—Athens Insomnia Scale; PSS-10—Perceived Stress Scale; *n*-number of, %-percent.

**Table 4:** Correlations between the respondents’ age and their psychosocial functioning (anxiety, depression, insomnia, stress).

Psychosocial Functioning Parameters	Pearson’s r	t	*p*
AIS	−0.11	−1.99	0.048
GAD-7	−0.06	−1.061	0.289
PHQ-9	−0.16	−2.907	0.004
PSS-10	0.01	0.155	0.877

GAD-7—Generalized Anxiety Disorder-7; PHQ-9—Patient Health Questionnaire-9; AIS—Athens Insomnia Scale; PSS-10—Perceived Stress Scale; t-Student’s t-distribution, *p*—significance level.

**Table 5:** The psychosocial functioning (anxiety, depression, insomnia, and stress) of the respondents depending on their sex.

Variables	Women		Men		t	*p*
	M	SD	M	SD		
AIS	7.66	4.51	7.18	4.52	0.777	0.438
GAD-7	15.78	6.04	13.66	5.34	2.623	0.009
PHQ-9	18.71	6.38	16.75	6.00	2.270	0.024
PSS-10	7.40	1.49	6.90	1.92	2.328	0.021

M—mean; SD—standard deviation; t—Student’s t-distribution, *p*—significance level; GAD-7—Generalized Anxiety Disorder-7; PHQ-9—Patient Health Questionnaire-9; AIS—Athens Insomnia Scale; PSS-10—Perceived Stress Scale.

**Table 6:** The psychosocial functioning (anxiety, depression, insomnia, stress) of the respondents depending on their parental status.

Variables	Children						F	*p* *
	Yes		No		Pregnancy			
	M	SD	M	SD	M	SD		
AIS	6.94	4.19	8.16	4.69	7.63	5.63	2.955	0.054
GAD-7	14.67	5.81	16.07	6.08	13.75	4.74	2.484	0.085
PHQ-9	17.14	5.98	19.53	6.54	16.25	5.09	6.201	0.002
PSS-10	7.30	1.68	7.31	1.52	7.00	1.51	0.141	0.868

M—mean; SD—standard deviation; F—the value of the test statistic; *p*—significance level; * one way ANOVA; GAD-7—Generalized Anxiety Disorder-7; PHQ-9—Patient Health Questionnaire-9; AIS—Athens Insomnia Scale; PSS-10—Perceived Stress Scale.

**Table 7:** Correlations between the parameters of psychosocial functioning.

Variables		Pearson’s r	t	*p*
GAD-7	AIS	0.53	11.24	<0.001
	PSS-10	0.50	10.236	<0.001
PHQ-9	GAD-7	0.76	21.14	<0.001
	AIS	0.65	15.35	<0.001
	PSS-10	0.45	8.919	<0.001
PSS-10	AIS	0.32	6.129	<0.001

GAD-7—Generalized Anxiety Disorder-7; PHQ-9—Patient Health Questionnaire-9; AIS—Athens Insomnia Scale; PSS-10—Perceived Stress Scale; *p*—significance level.

## Data Availability

Data sharing not applicable.

## Supplementary Materials

The following supporting information can be downloaded at: https://www.mdpi.com/article/10.3390/medicina58020196/s1. Table S1: The influence of education on the levels of anxiety, depression, perceived stress, and insomnia among the residents of the West Pomeranian Voivodeship during the SARS-CoV-2 pandemic. Table S2: The influence of place of residence on the levels of anxiety, depression, perceived stress, and insomnia among the residents of the West Pomeranian Voivodeship during the SARS-CoV-2 pandemic. Table S3: The influence of marital status on the levels of anxiety, depression, perceived stress, and insomnia among the residents of the West Pomeranian Voivodeship during the SARS-CoV-2 pandemic.
