# Supplementary material for: Incidence and Sociodemographic Correlates of Psychological Health Problems among Residents of the West Pomeranian Voivodeship during the COVID-19 Outbreak
Source: Medicina (Kaunas). 2022 Jan 27;58(2):196. doi: 10.3390/medicina58020196 (PMC8876927; doi:10.3390/medicina58020196)
Supplement: Supplementary file 1 [file medicina-58-00196-s001.zip › medicina-1501480-supplementary.pdf]

SUPPLEMENTARY MATERIALS

Table S1. The influence of education on the levels of anxiety, depression, perceived stress, and insomnia among the residents of the West Pomeranian Voivodeship during the SARS-CoV-2 pandemic.

Variables		Education					t	p
		secondary	post-secondary	higher (I)	higher (II)	higher (III)		
AIS	M	8.19	6.07	7.57	7.58	8.00	0.686	0.602
	SD	4.83	3.91	4.28	4.64	4.57		
GAD-7	M	7.45	5.86	8.20	8.87	10.33	1.580	0.179
	SD	5.90	4.61	5.93	6.05	5.99		
PHQ-9	M	9.09	7.24	8.66	9.97	10.33	1.116	0.349
	SD	6.66	7.26	5.86	6.41	6.80		
PSS-10	M	20.63	21.64	22.29	22.98	24.33	2.334	0.056
	SD	6.09	3.56	5.76	5.14	5.21		
M–mean; SD–standard deviation; t–Student’s t-distribution; p–significance level; GAD-7–Generalized Anxiety Disorder-7; PHQ-9–Patient Health Questionnaire-9; AIS–Athens Insomnia Scale; PSS-10–Perceived Stress Scale								

Table S2. The influence of place of residence on the levels of anxiety, depression, perceived stress, and insomnia among the residents of the West Pomeranian Voivodeship during the SARS-CoV-2 pandemic.

Variables		Place of residence				F*	p
		village	city with up to 10,000 people	city with 10,000-100,000 people	city with over 100,000 people		
AIS	M	7.45	7.69	7.43	7.70	0.087	0.967
	SD	4.44	3.72	4.60	4.64		
GAD-7	M	7.54	8.63	8.51	8.55	0.509	0.676
	SD	6.08	6.21	6.05	5.88		
PHQ-9	M	7.91	10.13	8.55	9.97	2.118	0.098
	SD	5.64	6.21	6.43	6.52		
PSS-10	M	21.58	23.38	22.64	22.45	0.688	0.560
	SD	5.41	4.72	6.27	5.37		
M–mean; SD–standard deviation; F– the value of the test statistic; p–significance level; *one way ANOVA GAD-7–Generalized Anxiety Disorder-7; PHQ-9–Patient Health Questionnaire-9; AIS–Athens Insomnia Scale; PSS-10–Perceived Stress Scale							

Table S3. The influence of marital status on the levels of anxiety, depression, perceived stress, and insomnia among the residents of the West Pomeranian Voivodeship during the SARS-CoV-2 pandemic.

**Variables**		**Marital status**			**F***	**p**
		Formal relationship	Informal relationship	Single / divorced / widowed		
**AIS**	**M**	7.16	7.62	8.33	1.903	0.151
	**SD**	3.99	4.66	5.18		
**GAD-7**	**M**	8.21	8.36	8.53	0.085	0.918
	**SD**	5.79	5.99	6.23		
**PHQ-9**	**M**	8.46	10.08	10.05	2.607	0.075
	**SD**	5.78	6.47	6.98		
**PSS-10**	**M**	22.39	22.92	21.79	0.895	0.410
	**SD**	5.88	4.87	5.37		

M–mean; SD–standard deviation; F– the value of the test statistic; p–significance level; *one way ANOVA; GAD-7–Generalized Anxiety Disorder-7; PHQ-9–Patient Health Questionnaire-9; AIS–Athens Insomnia Scale; PSS-10–Perceived Stress Scale
